# The moderating effect of emotion regulation in the association between social support and religiosity and psychological distress in adults

**DOI:** 10.1186/s40359-023-01160-z

**Published:** 2023-04-17

**Authors:** Joya-Maria Karam, Feten Fekih-Romdhane, Mirna Fawaz, Diana Malaeb, Sahar Obeid, Souheil Hallit

**Affiliations:** 1 School of Medicine, Lebanese University, Hadat, Lebanon; 2 The Tunisian Center of Early Intervention in Psychosis, Department of Psychiatry “Ibn Omrane”, Razi Hospital, Manouba, 2010 Tunisia; 3 Faculty of Medicine of Tunis, Tunis El Manar University, Tunis, Tunisia; 4 Faculty of Health Sciences, Beirut Arab University, Tareek Al Jadida, Afeef Al Tiba, Beirut, 1105 Lebanon; 5 College of Pharmacy, Gulf Medical University, P.O. Box: 4184, Ajman, United Arab Emirates; 6 School of Pharmacy, Lebanese International University, Beirut, Lebanon; 7 Social and Education Sciences Department, School of Arts and Sciences, Lebanese American University, Jbeil, Lebanon; 8 School of Medicine and Medical Sciences, Holy Spirit University of Kaslik, P.O. Box 446, Jounieh, Lebanon; 9 Applied Science Research Center, Applied Science Private University, Amman, Jordan; 10 Research Department, Psychiatric Hospital of the Cross, Jal Eddib, Lebanon

**Keywords:** Emotional regulation, Cognitive reappraisal, Suppressive expression, Social support, Religiosity, Psychological distress, Lebanon

## Abstract

**Background:**

Lebanese adults face daily obstacles due to their numerous responsibilities and non-ending external pressures to the extent that Lebanon has been ranked second among highest negative experiences countries worldwide. A sparse number of international studies showed that positive social support, religiosity and cognitive reappraisal would decrease psychological distress, but none in Lebanon. This study aimed to evaluate the association between social support, religiosity and psychological distress among Lebanese adults, taking into consideration the moderating role of emotion regulation.

**Methods:**

387 adult participants enrolled in this cross-sectional study between May and July 2022. Participants were chosen from five different governorates in Lebanon, using the snowball sampling technique, and were asked to complete a structured questionnaire, which included the following scales: the Mature Religiosity Scale, the Emotional Regulation Scale, the Depression Anxiety Stress Scale, and the Multidimensional Scale of Perceived Social Support.

**Results:**

The interaction social support by cognitive reappraisal was significantly associated with psychological distress; at high levels of cognitive reappraisal and low levels of expressive suppression, higher social support (Beta = − 0.07; *p* = .007) was significantly associated with lower psychological distress. The same was found at high levels of cognitive reappraisal and moderate levels of expressive suppression (Beta = − 0.08; *p* = .021). Social support alone was not significantly associated with psychological distress in the model (Beta = 0.15; t = 1.04; p = .300; 95% CI − 0.14; 0.44).

**Conclusion:**

This cross-sectional study has provided evidence that the adequate use of emotional regulation skills such as high level of cognitive reappraisal and low level of expressive suppression with presence of social support would remarkably decrease psychological distress. This result casts a new light on clinical approaches to tackle this association between the emotional regulation of a patient in interpersonal psychotherapy.

## Introduction

Distress is largely prevalent in adulthood [1]. It is manifested through several forms to suggest symptoms of depression (sadness, irritability, insomnia…) and anxiety (restlessness, fatigue…). Distress is recognized as a subjective experience influenced by negative life stressors such as loss of a job, financial obligations and chronic illnesses [2]. There is a growing concern about the development of its symptoms into a serious diagnosed illness [3]. Psychological distress ought to be counteracted by protective factors to preserve someone’s well-being. In this line, there is substantial evidence from recent studies showing that strong support system, satisfactory financial state, feeling of belonging and adequate self-reflection would decrease distress levels [4, 5].

In the United States, a previous study has given serious attention to the impact of social support analyzing three of its pillars: family, partner and friends’ relationships. In a population of 602 Latinos, there was a 43% and 31% decrease in depressive symptoms due to family and friend support respectively; partner support successfully alleviated distress as well [6]. Moreover, based on empirical literature analysis, it is agreed that interpersonal networking is a fundamental human need promoting a sense of belonging [7]. Over and above that, studies conducted during the COVID-19 pandemic reported a reduction in traumatic stress (patients’ death, viral exposures…) experienced by health-care workers when support was procured by colleagues and family [8, 9]. Furthermore, literature suggests that men are less likely to seek social support than women; therefore, they are at higher risk of psychological distress [10].

In addition, studies shed the light on the correlation between religiosity and less psychological distress. Religiosity is valued by a large portion of the population; it is interpreted and understood differently worldwide but provides a common sense of self-esteem, belongingness and comfort, which enhances the mental health state [11]. For example, a significantly large study by Gallop Polls in 143 countries revealed that 92% of people in developing countries stated that religiosity is “an important part of their daily life”. This study went beyond this finding by also reporting that 65% of the religious population are more likely to feel enjoyment than people in the less religious group. The more vulnerable the community is (e.g., Uganda, Ethiopia and Burkina Faso), the more likely its people seek religiosity for hope, which would positively affect their mental health. As they describe it in their study, it was highly indicative that religiosity promotes the feeling of belongingness and “social security” against negative emotions [12]. However, it is noteworthy that other findings suggested that religiosity has a weak link or even no correlation with reducing symptoms of anxiety and stress [13].

Furthermore, psychological distress is connected to one’s capacity to understand and assess the situation given at hand and consider it as a challenging or stressful situation [13]. The two major emotional regulation skills are [1] cognitive reappraisal, which is the attempt to reinterpret the internal emotions during a stressful event and [2] expressive suppression, defined as a repression of one’s emotion and inhibition of any facial expression that could reflect them [14].

De facto, a series of study clearly stated that difficulties in regulating emotions led to symptoms of anxiety, and violent behavior [14–16]. Moreover, common findings agreed that, compared to women, men were more prone to use expressive suppression by hiding their emotions from others [17]. In addition, a sample of 315 students at Zanjan Univeristy reported an increase in anxiety and depression in people who did not regulate their emotions by 53% and 66% respectively [18]. In the same line, a study suggested that adolescents who master emotional regulation are happier than those who do not [19].

In this context, social support develops one’s self-esteem and self-confidence to be able to overcome stress. The results of a study conducted among 507 fishermen who are exposed to daily ocean trips, reported that those who were supported by their local peers and neighbors had good emotion regulation. This is because their peers would discuss the good outcomes of their trip with them, while letting them express their emotions (and not suppress them), thus reducing their psychological distress [20].

In only one study of 203 young adult participants, religiosity was elucidated as a protective factor that promotes better cognitive reappraisal, self-esteem, control and resilience; this would prevent anxiety and depression and consequently decrease the risk of psychological distress [21].

**Fig. 1:**
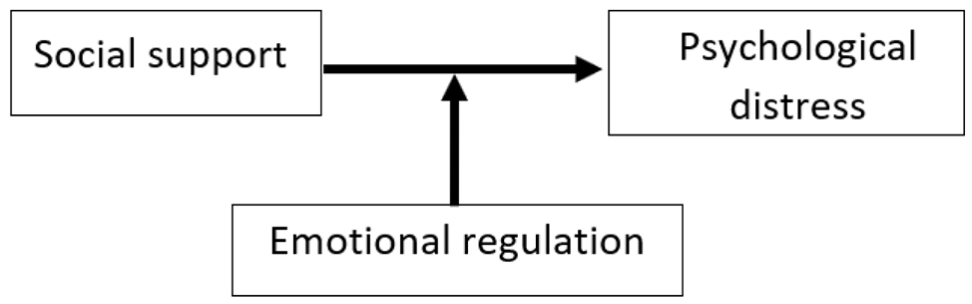
The moderating effect of emotional regulation in the association between social support and psychological distress

However, little is known about the association between social support and religiosity on emotional regulation. We acknowledge that there is a single study [22], conducted among senior citizens that showed that high cognitive reappraisal, in the presence of social support, would lessen the negative symptoms of depression in elderly people (see Fig. 1). This study also explained that emotional regulation should serve as an interpersonal problem-solving strategy in order to have more robust social networks and consequently decrease stress and negative experiences [22]. Researchers found that the combination of high reappraisal and high religious coping are beneficial protective strategies against negative emotions in young adults [21].

### Significance of the study

Lebanon, a Middle Eastern country, is crippled by multiple crises with threatening consequences on the population’s mental health. On August 4, 2020, Beirut has been a victim of the World’s most powerful non-nuclear explosion in the 21th century, which led to the death of more than 200 people and the injury of more than 6000, while leaving thousands of people homeless [23]. This tragedy has been aggravated by the fact that Lebanese people are paid the lowest minimum wage worldwide, which is a limitation for them to adapt against the economic inflation of 154.8% [24] and be able to provide the bare minimum for their families (food, price of rent, pay bills…). A study in 2021 showed that those whose income decreased by 75% would experience fear of poverty, exposing them to high levels of anxiety and stress [25]. Revealing the multiple psychological consequences of these innumerable events would be very complex; Embrace, a national non-governmental association, revealed that 67% out of 2,239 calls on the suicide prevention lifeline suffered from emotional distress, were always sad and had no pleasure to pursue their usual activities, whereas 28% mentioned having suicidal ideations following the Beirut blast [26].

On the other hand, Lebanon is a small country known for its “collectivist culture” [27, 28] where all family members are involved in each other’s life. In the Middle East region, the collectivist culture comes with the exchange of connectedness that pushes people to talk more about their emotions and abstain from “expressive suppression” [29]. In line with that, Arab people fear any harm that could affect their family members or their reputation, which could explain the investment in each other’s lives [30].

**Fig. 2:**
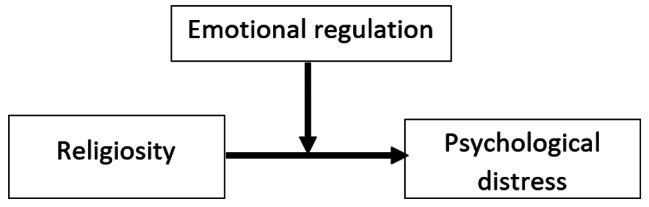
The moderating effect of emotional regulation in the association between religiosity and psychological distress

Moreover, religiosity in the Middle East countries is considered as fostering positive well-being [31]. In Lebanon, religious people often attribute negative experiences to a spiritual intervention, as a punishment from God [30]. Yet, they try to seek forgiveness from God by implementing good qualities such as faith, patience and optimism as seen in Fig. 2 [32]. Lebanese participants with high negative religious coping had lower mental QOL despite having positive experiences in life [33].

Therefore, this study aimed to assess the relationship between social support, religiosity and psychological distress, taking into consideration the moderating role of emotion regulation. We expected that social support alone or religiosity alone do not contribute to decrease psychological distress among Lebanese adults. We also hypothesized that higher social support and religiosity among Lebanese adults would be correlated with lower psychological distress, being moderated by higher cognitive reappraisal.

## Methods

### Study design

A cross-sectional study was carried out between May and July 2022, enrolling 387 participants, and using an anonymous, self-administered questionnaire created on Google forms. To reach the largest possible group of subjects, the research team initiated the contact with friends and family members they know; those people were asked to forward the link to their friends and family members, and were asked to forward the link to their contact list via social media applications such as WhatsApp, Facebook Messenger, and Instagram. Hence, the link was shared among the participants and sent to all districts/governorates of Lebanon (Beirut, Mount Lebanon, North/Akkar, South/Nabatiyeh, and Bekaa/Baalback-Hermel) through social networks, using the snowball technique. Before obtaining the informed consent, the participants were notified about the objective of the study and assured of the anonymity of the response. Participants had the right to enroll in this study without any obligation or pressure from the research team with no monetary compensation given to them for participation. All subjects above 18 years were eligible to participate and were asked to send the link to other subjects. Excluded were those who refused to fill out the questionnaire.

### Minimal sample size calculation

We used G*Power software to determine the sample size. The minimum required sample size was 226 participants, considering an alpha error of 5%, a power of 90%, a minimal model r-square of 10% and allowing 15 predictors to be included in the model.

### Questionnaire

Lebanese participants were asked to fill in an anonymously designed survey questionnaire in their native language (Arabic) that required approximately 20 min. The first part of the questionnaire included an explanation of the study objective, and a statement ensuring the anonymity of respondents. The participants had to select the option stating *I consent to participate in this study* to be directed to the questionnaire.

The second part of the questionnaire contained sociodemographic information about the participants (age, sex, region of living, marital status and education level). The Household Crowding Index (HCI), reflecting the socioeconomic status of the family [34], is the ratio of the number of persons living in the house over the number of rooms in it (excluding the kitchen and the bathrooms).

The third part included the scales used in this study:

#### DASS-8 (depression anxiety stress Scale- 8 items)

The DASS-8 was used to screen psychological distress symptoms (depression, anxiety and stress). Each item is rated over a 4- point scale from 0 (did not apply to me at all) to 3 (applied to me very much). The total score of DASS-8 ranges from 0 to 24; the higher the score the higher the presence of mental symptoms. It has been previously validated in the Middle Eastern region [35] (McDonald’s omega = 0.90 in this study and 0.87 in the original study).

#### Multidimensional Scale of Perceived Social Support (MSPSS)

This tool includes 12 items divided into three major sources of social support [36]: family [4], friends [4] and others [4]. This Persian version of the MSPSS adopted a 5- point Likert scale (0 = strongly disagree, 5 = strongly agree). Higher scores indicated higher social support (McDonald’s omega = 0.97 in this study; Cronbach’s alpha = 0.88 in the original study) [39]. We used the Arabic version already validated in Lebanon [37].

#### Mature religiosity scale (MRS)

This tool includes 16 criteria that are unambiguous and simple to assess the faith of a person [38]. The scale is standardized as 1 (totally agree) and 5 (totally disagree). Moderate scores indicated high religiosity from time to time (rituals practicing and church attendance, reading the bible) and higher scores indicated being actively religious on a regular basis. The Arabic version of the scale has been used previously [38] (McDonald’s omega = 0.97 in this study; Cronbach’s alpha = 0.92 in the original study) [38].

#### Emotional regulation questionnaire

Validated in Lebanon [39], the Emotional Regulation Questionnaire is designed to evaluate two strategies used to regulate one’s emotions, which are cognitive reappraisal and expressive suppression. This questionnaire consists of 10 items each assessed by a 7- point Likert type scale from 1 (strongly disagree) to 7 (strongly agree). Higher scores reflect a larger use of the concerned emotion regulation strategy [40] (McDonald’s omega = 0.91 for cognitive reappraisal and 0.84 for expressive suppression in this study; in the original study, the Cronbach’s alpha values varied between 0.75 and 0.82 for cognitive reappraisal and 0.68-0.76 for expressive suppression) [41].

### Statistical analysis

The SPSS software v.25 was used for the statistical analysis. The psychological distress score was considered normally distributed since the skewness (= 0.096) and kurtosis (=-0.252) values varied between − 1 and + 1 [42]. The Student t was used to compare two means and the Pearson test was used to correlate two continuous variables. The moderation analysis was conducted using PROCESS MACRO (an SPSS add-on) v3.4 model 1 [43], taking social support and mature religiosity scores as independent variables, cognitive reappraisal/expressive suppression as moderators and psychological distress as the dependent variable. Results adjusted over age, sex, marital status, education level and household crowding index. *P* < .05 was deemed statistically significant.

## Results

### Sociodemographic and other characteristics of the sample

Three hundred eighty-seven participants participated in this study, with a mean age of 26.17 ± 11.47 years and 58.4% females. Other descriptive statistics of the sample can be found in Table 1.

**Table 1:** Sociodemographic and other characteristics of the sample (N = 387)

Variable	N (%)
Sex	
Male	161 (41.6%)
Female	226 (58.4%)
Marital status	
Single	311 (80.4%)
Married	76 (19.6%)
Education level	
Secondary or less	66 (17.1%)
University	321 (82.9%)
Region of living	
Urban	294 (76.0%)
Rural	93 (24.0%)
	**Mean ± SD**
Age (years)	26.17 ± 11.47
Household crowding index (persons/room)	1.47 ± 1.00

### Bivariate analysis of factors associated with psychological distress

The results of the bivariate analysis of factors associated with psychological distress are summarized in Tables 2 and 3. The results showed that none of the variables was significantly associated with psychological distress.

**Table 2:** Bivariate analysis of factors associated with psychological distress

Variable	Psychological distress (mean ± SD)	*P*
Sex		0.641
Male	5.99 ± 0.47	
Female	12.09 ± 6.30	
Marital status		0.695
Single	12.15 ± 6.26	
Married	12.46 ± 5.80	
Education level		0.054
Secondary or less	13.42 ± 5.38	
University	11.96 ± 6.29	
Region of living		0.422
Urban	12.35 ± 6.10	
Rural	11.76 ± 6.39	

**Table 3:** Correlations of continuous variables with psychological distress

	1	2	3	4	5	6	7
1. Psychological distress	1						
2. Social support	− 0.04	1					
3. Mature religiosity	0.05	0.61***	1				
4. Cognitive reappraisal	0.002	0.59***	0.47***	1			
5. Expressive suppression	− 0.05	− 0.46***	− 0.41***	− 0.74***	1		
6. Age	0.02	− 0.03	− 0.03	− 0.03	0.01	1	
7. Household crowding index	− 0.02	− 0.04	0.07	− 0.08	0.09	0.13*	1

*p < .05; ***p < .001

### Moderation analysis with psychological distress taken as the dependent variable

The details of the moderation analysis of cognitive reappraisal/expressive suppression taken as moderators in the associations between social support/mature religiosity and psychological distress, are summarized in Table 4. The interaction social support by cognitive reappraisal was significantly associated with psychological distress (Table 4). At high levels of cognitive reappraisal and low levels of expressive suppression, higher social support (Beta = − 0.07; *p* = .007) was significantly associated with lower psychological distress (Table 5). The same was found at high levels of cognitive reappraisal and moderate levels of expressive suppression (Beta = − 0.08; *p* = .021). It is noteworthy that social support alone was not significantly associated with psychological distress in the model (Beta = 0.15; t = 1.04; p = .300; 95% CI − 0.14; 0.44).

**Table 4:** Moderation analysis taking social support/mature religiosity as independent variables, cognitive reappraisal/expressive suppression as moderators and psychological distress as the dependent variable

Moderator	Beta	T	*P*	95% CI
**Model 1: Social support as an independent variable**				
Cognitive reappraisal	− 0.007	-2.06	0.040	− 0.013; − 0.001*
Expressive suppression	− 0.08	− 0.27	0.791	− 0.63; 0.48
**Model 2: Mature religiosity as an independent variable**				
Cognitive reappraisal	− 0.003	− 0.55	0.585	− 0.01; 0.01
Expressive suppression	0.001	0.14	0.888	− 0.01; 0.02

*indicates significant moderation; results adjusted over age, sex, marital status, education level and household crowding index

**Table 5:** Conditional effects of the focal predictor (social support) at values of the moderators

Cognitive reappraisal	Expressive suppression	Beta	*t*	*p*	95% CI
Low (= 15.47)	Low (= 11.05)	0.04	0.71	0.479	− 0.07; 0.14
Low (= 15.47)	Moderate (= 16.56)	0.03	0.97	0.335	− 0.03; 0.10
Low (= 15.47)	High (22.07)	0.03	1.00	0.318	− 0.03; 0.08
Moderate (= 23.84)	Low (= 11.05)	− 0.02	− 0.54	0.591	− 0.08; 0.05
Moderate (= 23.84)	Moderate (= 16.56)	− 0.02	-1.17	0.244	− 0.06; 0.02
Moderate (= 23.84)	High (22.07)	− 0.03	− 0.90	0.371	− 0.09; 0.04
High (= 32.20)	Low (= 11.05)	− 0.07	-2.69	**0.007**	− 0.13; − 0.02
High (= 32.20)	Moderate (= 16.56)	− 0.08	-2.32	**0.021**	− 0.15; − 0.01
High (= 32.20)	High (22.07)	− 0.09	-1.57	0.117	− 0.19; 0.02

Numbers in bold indicate significant *p* values

## Discussion

Up to date, this is the first study conducted in Lebanon that found interest in the association between emotional regulation, social support, religiosity and psychological distress. Findings of the current study showed that social support alone was not significantly associated with psychological distress. Interestingly, our results are in line with a previous study that strongly showed that despite the assumption of Lebanon having robust family ties as a prime support system, we do not find appropriate quality of support that would remarkably decrease the distress level of Lebanese population [27]. Moreover, another evaluation of social support in Lebanon came to the conclusion that social network was not conducive to decrease psychological distress when individuals lack trust in others especially in older adults’ group [44]. In contrast, a survey conducted in Netherlands suggested a positive correlation between perceived social support and mental health state [45].

Results of our study showed that at high levels of cognitive reappraisal and low or moderate levels of expressive suppression, higher social support was significantly associated with lower psychological distress, in line with a survey conducted among older adults (mean age of 69.46 years) in North Florida demonstrating that emotional regulation is associated with higher levels of perceived social support as a “buffering effect” against negative experiences of depression [22]. On top of that, cognitive reappraisal is a good skill acquired with age to preserve interpersonal relationships and attenuate the negative impact of stress [22]. In other words, anthropologists have come to realize that Lebanese people are innately collectivists [28]. Although people who live ingroup are more likely to receive social support [28], it would not be of remarkable benefit in order to decrease Lebanese people’s distress since the presence of peer family member was always available. In short, social interactions alone and emotional regulation skills alone would not be helpful tools against cumulative stressors in Lebanon as it has been ranked 2nd among the highest negative experiences’ countries in 2022 by Gallup’s annual reports [46]. From this standpoint, having both high cognitive reappraisal and low expressive suppression with good social support would successfully participate in lowering psychological distress levels; this has recently been conclusive in a study in Lebanon showing that positive emotional regulation would serve to increase resilience [47]. As a living proof of our findings, Embrace, a Lebanese non-governmental organization, has counted that more than 94% of people who call the lifeline to talk about their stressful life specifically in Lebanon would deescalate their level of distress; this supports our findings and proves that encouraging low expressive suppression and seeking social support are able to decrease psychological distress [48].

Furthermore, our results did not show that emotion regulation skills play a moderating role between religiosity and psychological distress. When comparing our results with those of older studies, it must be pointed out that religiosity is sometimes weakly correlated to emotional regulation in order to alleviate psychological distress [13]. Religiosity is one of the prominent values of culture in Lebanon; for believers, breaking God’s rules is often accompanied by shame and feelings of emotional turmoil and even a sense of guilt. Since the Arab World lives by conservative rules regarding family’s honor [49], it would be of possible explanation that Arab people would turn to the family network to alleviate mental health distress rather than seeking religious help.

Oppositely, our study also shows that religiosity has a direct effect on psychological distress; this has been previously evaluated in Lebanon by previous studies [21, 50, 51] demonstrating that religiosity would give a positive meaning to one’s life and encourage the individual to have better control over his emotions and behaviors. A study conducted in Lebanon among 333 young adults concluded that positive religious coping works towards decreasing people’s mental distress and improving their quality of life [52]. Another research conducted in Lebanon showed that 16% of people who practiced religious activities but were living in poor areas (such as Burj Barajneh camp, Nabaa and Hay El Selloum) suffered from depression [53]. These various studies show that effect of religiosity on decreasing psychological distress in Lebanon is different from a population to another. This might be due to the severity of bad experiences lived by the individual [54] or even due to very mediocre socioeconomic background that even religiosity cannot seem to alleviate.

In a country full of adversities and political, medical, educational events, our study shed the light on uncharted ways as cognitive reappraisal to make religiosity and social support be perceived as protective factors of mental health; those results might fill the gaps expressed in a recent study conducted in Lebanon that did not offer extensive explanations on coping mechanisms [55].

### Clinical implications

The study’s findings would suggest the importance of implementing specific techniques such as Mindfulness Based Cognitive Therapy (MBCT), recognized to be a fruitful tool to help build emotional regulation strategies such as cognitive reappraisal to decrease the level of distress [50]. In addition, interpersonal psychotherapy was a field of interest to many researchers. In fact, they were keen to demonstrate that fortifying intersocial relationships would contribute to relieving symptoms of psychological distress among elderly [51], university students [56], post-partum women [57] and even HIV patients [58]. Unfortunately, the development of emotional regulation skills conducive to a positive perception of social support has not been yet studied in the literature. This investigation would be beneficial because social support is a crucial factor for improving mental health of many patients. Moreover, these findings are consistent with another research showing that cognitive behavioral therapy has to provide the patient with tools encouraging him to avoid suppressing his emotions; it is important for the individual to perceive his social network as a space to share his emotions in order to decrease his level of distress [59].

### Limitations

First, the data was collected through an online survey, which might produce response bias. Second, the current study is cross-sectional and therefore causation cannot be inferred regarding the relationship between variables. Third, information bias may result from the use of self-report measures; participants may have overstated or underestimated some questions, introducing subjectivity in responding to questions. Finally, because other variables that may influence the psychological distress were not evaluated in this study, the likelihood of residual confounding bias must be mentioned.

## Conclusion

This cross-sectional study has provided evidence that the adequate use of emotional regulation skills such as high level of cognitive reappraisal and low level of expressive suppression with presence of social support would remarkably decrease psychological distress. This result casts a new light on clinical approaches to tackle this association between the emotional regulation of a patient in interpersonal psychotherapy. However, the interaction religiosity by emotion regulation was not significantly associated with psychological distress; further studies should analyze the conditions under which religiosity can effectively decrease psychological distress.

## Data Availability

All data generated or analyzed during this study are not publicly available due to the restrictions by the ethics committee (data are owned by the Psychiatric Hospital of the Cross). The dataset supporting the conclusions is available upon request to Ms. Rana Nader (rnader@naderlawoffice.com), a member of the ethics committee at the Psychiatric Hospital of the Cross.
